# Improving flood hazard datasets using a low-complexity, probabilistic floodplain mapping approach

**DOI:** 10.1371/journal.pone.0248683

**Published:** 2021-03-29

**Authors:** Rebecca M. Diehl, Jesse D. Gourevitch, Stephanie Drago, Beverley C. Wemple

**Affiliations:** 1 Department of Geography, University of Vermont, Burlington, Vermont, United States of America; 2 Gund Institute for Environment, University of Vermont, Burlington, Vermont, United States of America; 3 Rubenstein School of Environment and Natural Resources, University of Vermont, Burlington, Vermont, United States of America; Bristol University/Remote Sensing Solutions Inc., UNITED STATES

## Abstract

As runoff patterns shift with a changing climate, it is critical to effectively communicate current and future flood risks, yet existing flood hazard maps are insufficient. Modifying, extending, or updating flood inundation extents is difficult, especially over large scales, because traditional floodplain mapping approaches are data and resource intensive. Low-complexity floodplain mapping techniques are promising alternatives, but their simplistic representation of process falls short of capturing inundation patterns in all situations or settings. To address these needs and deficiencies, we formalize and extend the functionality of the Height Above Nearest Drainage (i.e., HAND) floodplain mapping approach into the probHAND model by incorporating an uncertainty analysis. With publicly available datasets, the probHAND model can produce probabilistic floodplain maps for large areas relatively rapidly. We describe the modeling approach and then provide an example application in the Lake Champlain Basin, Vermont, USA. Uncertainties translate to on-the-ground changes to inundated areas, or floodplain widths, in the study area by an average of 40%. We found that the spatial extent of probable inundation captured the distribution of observed and modeled flood extents well, suggesting that low-complexity models may be sufficient for representing inundation extents in support of flood risk and conservation mapping applications, especially when uncertainties in parameter inputs and process simplifications are accounted for. To improve the accuracy of flood hazard datasets, we recommend investing limited resources in accurate topographic datasets and improved flood frequency analyses. Such investments will have the greatest impact on decreasing model output variability, therefore increasing the certainty of flood inundation extents.

## 1. Introduction

Globally, runoff and streamflow patterns are shifting as a result of climate change [1,2]. Human populations often concentrate along rivers and their floodplains because of opportunities for commerce, transportation, and agriculture [3] and this trend is exacerbated as populations grow, translating to a growing threat to human life and property [4]. In light of these increasing hazards, it is imperative to effectively communicate flood risks to help save lives and protect property [5]. Furthermore, floodplains that are regularly inundated by flood waters benefit humans and river ecosystems, reducing downstream flood damages, increasing water quality, and providing aquatic and riparian habitat [6,7]. Thus, floodplain functioning depends on the degree to which it is connected to its river channel.

Traditionally, planners, policy makers, scientists, and conservation professionals have relied on deterministic floodplain maps that depict the extent of flood inundation. In the United States, the Digital Flood Insurance Rate Maps (DFIRM), an extensive dataset developed in support of the National Flood Insurance Program (NFIP), demarcates the boundary between areas flooded during the 100-year event (i.e., corresponding to a discharge with a 1% annual exceedance probability), and those that remain dry. These floodplain maps are widely referenced for floodplain planning and insurance assessments [8] and also inform conservation and restoration initiatives [9].

Numerous studies have suggested that the accuracy of DFIRM maps are limited and that for some communities the information is outdated or missing altogether [5,10–12]. Uncertainties in data inputs and poor representation of hydrologic and hydraulic processes contribute to discrepancies between existing maps and recent observations [13,14]. Hydrologic uncertainties, already large [15], are likely to continue to grow with a changing climate [16], further degrading the utility of deterministic floodplain maps. Traditional techniques used to create DFIRM maps, including 1D and 2D hydraulic models, are data intensive and therefore expensive, making it infeasible to efficiently correct map inaccuracies and fill gaps in coverage.

In response to the growing sense of urgency for new flood risk products, numerous floodplain mapping approaches have been proposed and a new suite of models have emerged [e.g., 11,17,18]. These models represent a simplified alternative to traditional hydraulic/hydrodynamic models. Traditional models account for the physics of water flow through a channel and onto the floodplains and require significant detail to describe the underlying terrain and hydraulic conditions of the stream channel and floodplain. Many of the alternative approaches simplify the representation of process [19–21] or of underlying terrain [17], making their application over large spatial scales possible [22,23]. Such frameworks hold promise for improving flood risk planning and emergency response as well as for conservation applications [21,24].

The application of simplified floodplain models are not without challenge [20]. Most notably, rapidly expanding model domains necessitates identifying values for an increasing number of parameters, and calibration datasets are often not available. Although improvements to high resolution topography and other remote sensing products provides for rapid, and extensive measurements of many model inputs, these datasets are error-prone and uncertain [14]. Large uncertainties in input parameters and simplified representations of process can interact and propagate, decreasing map accuracy, notably where topography is complex or backwater influences large [20]. Accounting for the various sources of uncertainty will thus improve upon low-complexity model outputs. Yet, because of the large number of input parameters needed for large areas, and numerous sources of uncertainty, questions remain as to where to invest limited human and financial resources to improve on model output [25].

In this paper, we describe a relatively rapid, probabilistic approach to delineating floodplains defined by the maximum extent of a flood of a given recurrence interval (i.e., the hydraulic floodplain). Representation of flood boundaries as bands of probabilities communicates risks, and related uncertainties, associated with flooding based on the current understanding of relevant conditions and processes [26,27]. Our approach accounts for uncertainty associated with measurement error and simplification of process. To do this, we incorporate a Monte Carlo simulation into the lower-complexity height above nearest drainage (i.e., HAND) modeling approach described by Zheng et al. [28], hereafter called the probHAND model.

Although a HAND-based probabilistic approach has recently been proposed [29], ours differs in a few important ways. Jafarzadegan and Merwade [29]’s approach uses existing DFIRM maps as reference, which are wrought with errors and uncertainty [5]. In addition, their approach does not allow for hydraulic or hydrologic variable specification. As such, one cannot use their model to evaluate how inundation may shift with changing inputs, such as from a changing climate or land use [30].

In the following we first describe the probHAND model, including the workflow and required inputs (Section 2). In Section 3, we then demonstrate application of the probHAND model in the Vermont portion of the Lake Champlain Basin. We describe the specific inputs for our application. The integrity of the rapid, large-scale approach to mapping is compared with a more complex hydraulic modeling approach (i.e. 1D HEC-RAS) for a single watershed, as well as with surveyed flood extents. Because large model domains involve considerable parameterization, it is difficult to accurately constrain all inputs, especially with limited financial and human resources [25,31]. We explore the value of information for probabilistic floodplain maps by calculating the input parameters’ contribution to model output variance and evaluate how the size and shape of the PDF and the valley setting influences the relative influence of each input on the variability of model output. Our results demonstrate the value of simple probabilistic floodplain mapping approaches and have important implications for model-based flood risk management and conservation.

## 2. ProbHAND model

**Fig 1:**
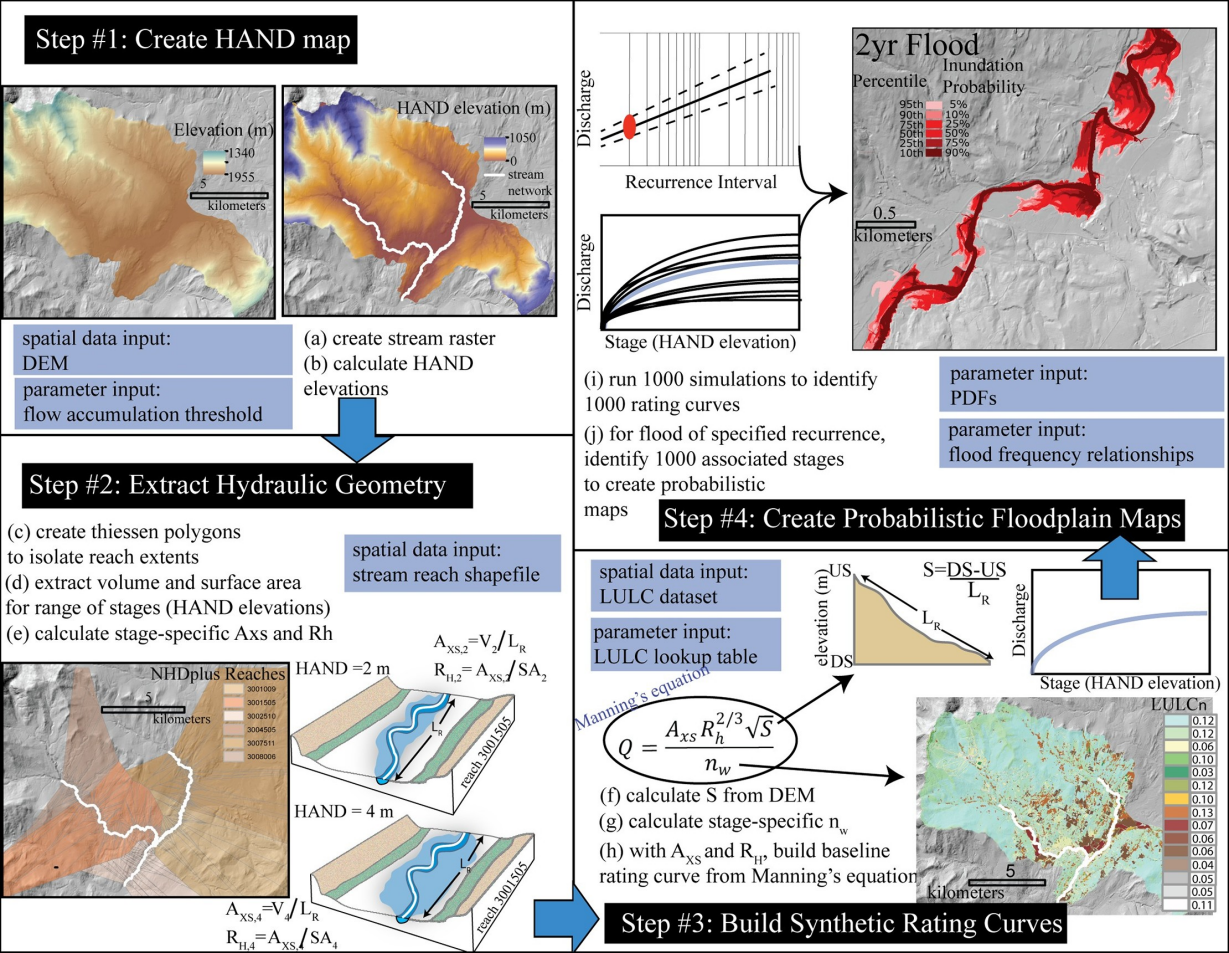
Model workflow. Execution of probHAND model includes four steps: 1) create HAND map from DEM, 2) extract hydraulic geometry from HAND map for each reach, for a range of water stages which correspond to HAND elevations, 3) build synthetic rating curves to relate stage to discharge, and 4) translate rating curve to probabilistic floodplain map using flood frequencies, considering uncertainties in input parameters used to develop synthetic rating curves and identify flood frequencies. Input datasets and variables are highlighted by blue boxes. Spatial datasets in figure are from the University of Vermont’s Spatial Analysis Lab.

The probHAND model is 1) easy to implement, requiring input data that is increasingly available for most communities, 2) scalable, in order to map floodplains relatively rapidly over large areas, and 3) robust in its communication of flood extents and associated uncertainty. ProbHAND builds on the HAND mapping approach described by Zheng et al. [28] and adapted by others [32] including the National Water Model for flood forecasting [22]. Although the original HAND approach is relatively tractable to implement, it has not been formalized into an accessible model and its products are deterministic. We adopted and updated the original HAND approach to incorporate uncertainty and produce probabilistic floodplain maps. We implemented the probHAND model in Python v3.7, using exclusively open-source packages (i.e. not ArcPy). Below, we describe the four steps involved in the probHAND mapping approach (Fig 1). Model inputs are described in Table 1.

**Table 1:** General inputs for probHAND model, specifying inputs for the Lake Champlain Application.

Step	Spatial Data Inputs	Lake Champlain Application	Parameter Inputs	Lake Champlain Application	Parameter Uncertainty*	Lake Champlain Application
1	DEM	1 m LiDAR-derived hydro-flattened DEM from the State of Vermont	flow accumulation threshold	10 mi^2^		
2	Stream Reach Shapefile	NHDplus, streams with DA>10 mi^2^				
3	LULC Raster	1 m 2016 LULC for State of Vermont	*n* values associated with LULC	lookup table from Truehart (2019)	*A*_*XS*_, *R*_*H*_, *n*_*w*_, *S*	Uncertainty evaluated from USGS gages and HEC-RAS models**
4			RI-Flood Peak Curve	USGS Stream Stats (Olson 2002)	*Q*_*RI*_	Standard error of flood frequency regressions (Olson 2002)

*Represented by probability density functions. Distribution types may be chosen by user.

** See S1 Text.

### Step 1: HAND map creation

A Height Above Nearest Drainage (HAND) map is created from a digital elevation model (DEM), representing the elevation difference between each land surface cell and the stream bottom cell to which it drains [33]. To create the HAND map, probHAND applies a sequence of routing functions using TauDEM command-line tools [34]. The first tasks are to prepare the DEM for a hydrologic analysis, which includes removing obstacles (e.g., bridges) and filling pits (i.e., low spots in the DEM where water would pool). For the filled DEM, topography-driven flow direction is mapped using a D-infinity flow algorithm [35]. Next, the model creates a stream raster, where the location of the stream is represented by a “1” and all other cells are assigned “0”. The extent of the stream network is defined by a flow accumulation threshold. Using the DEM, flow direction, and stream rasters, the vertical distance between each cell and the nearest (based on the flow pathway) stream cell is calculated in TauDEM’s infinity distance down tool.

### Step 2: Extract hydraulic geometry

The probHAND model extracts average hydraulic geometry values from the HAND layer, on a reach-by-reach basis, in order to support the development of synthetic rating curves (see step 3). To extract reach-average hydraulic geometry values, it is first necessary to provide a polyline shapefile delineating stream reaches. The probHAND model uses this shapefile to create Thiessen polygons (also known as “Voronoi polygons”) from each node along the stream reach, merging all Thiessen polygons that have the same reach ID value. Using reach-scale polygons, the model calculates a volume (*V*_*y*_) in m^3^ and surface area (*SA*_*y*_) in m^2^ for successively increasing HAND values, or stages (*y*), above the channel bottom, by intersecting a plane with the HAND map. The model extracts hydraulic geometry for a large range of stages (from 0.01 m to 50 m) to assure inclusion of discharges of interest in highly variable topographic settings. For each reach, the probHAND model then calculates reach-average cross-sectional area (*A*_*XS*_) in m^2^ and hydraulic radius (*R*_*H*_) in m for each stage (*y*) based on the length of each reach along the streamline (*L*_*R*_),

AXS,y=VyLR [1]

$$R_{H,y}=A_{XS,y}/\frac{{SA}_{y}}{L_{R}}=V_{y}/{SA}_{y}$$ [2]

### Step 3: Build synthetic rating curves

The probHAND model uses the Manning’s equation to build synthetic rating curves for each reach, calculating a discharge for a given HAND value (i.e., stage above channel bottom). Thus, for each reach, at each stage, the model requires hydraulic geometry information (*A*_*xs*_, *H*_*radius*_; Eqs 1 and 2), the water surface slope (*S*), and a roughness coefficient (*n*_*w*_). The model approximates the value of *S*, as the channel slope, by extracting the stream elevation at the end and start of the reach from the DEM, and dividing by reach length [36]. The model uses a look-up table approach to calculate *n* for the reach, at each stage [37]. A land use and land cover (LULC) raster is required as input. Each LULC category (*l*) is assigned a unique *n* value (n_LULC,l_), which must be defined as an input, is weighted, *n*_*W*,*y*_, by the area within the reach in each LULC category (*A*_*LULC*_), for a given stage (*y*):

$$n_{W,y}=\frac{\sum_{l=1}^{N}n_{lulc,l}A_{l,y}}{\sum_{l=1}^{N}A_{l,y}}$$ [3]

### Step 4: Create probabilistic floodplain maps

Floodplain maps delineate the inundation extent associated with the peak discharge of a characteristic flood event (i.e., the 100-year flood). The probHAND model identifies the probability of inundation for floods with a recurrence interval that ranges between once every 2 and 500 years, specified by the peak discharge associated with that recurrence interval (*Q*_*RI*_). The model uses unique flood frequency information for each reach, associating a stage with *Q*_*RI*_ values from the rating curve developed in step 3. This initial stage, the associated values calculated by the probHAND model to define the rating curve (*A*_*XS*_, *R*_*H*,_ *S*, *n*_*w*_*)*, and the user-defined *Q*_*RI*_ values, are referred to as the baseline values.

Baseline values have uncertainties as a result of measurement error (*A*_*XS*_, *R*_*H*_), limited data (*Q*_*RI*_), or a general lack of information on hydraulic processes (*S*, *n*_*w*_) [38]. To account for this, the model performs a Monte Carlo-based uncertainty analysis. Probability density functions (PDFs) may be used to describe the uncertainty around baseline values of hydraulic geometry (*A*_*XS*_, *R*_*H*_), roughness coefficient (*n*_*W*_), energy grade slope (*S*), and the discharge for a given recurrence interval (*Q*_*RI*_). Both the type of distribution (e.g., https://docs.scipy.org/doc/scipy/reference/stats.html) and associated parameters for each variable may be defined. For each iteration of the Monte Carlo simulation (N = 1000), parameter values are randomly sampled from their fitted PDFs, and a unique rating curve is created. The model then draws 1000 observations of *Q*_*RI*_ from its PDF, which are matched with one of the 1000 stage-discharge rating curves. As a result, 1000 stages are identified for each recurrence interval flood. Stages are ranked from lowest (i.e., smallest HAND elevation) to highest (i.e., largest HAND elevation) and converted to percentiles, whereby small percentiles are associated with small HAND elevations which are predicted to be inundated for all or most of the 1000 possible scenarios, and therefore have a high probability of inundation for a specific flood event. Conversely, large percentiles are associated with large HAND elevations which include areas that only get inundated by a few of the scenarios, and therefore have a low probability of inundation for a specific flood event. The recurrence interval-probability-stage relationships are translated to floodplain percentile maps by intersecting the stage (HAND elevation) with the HAND map on a reach-by-reach basis.

## 3. Application to the Lake Champlain Basin, Vermont

**Fig 2:**
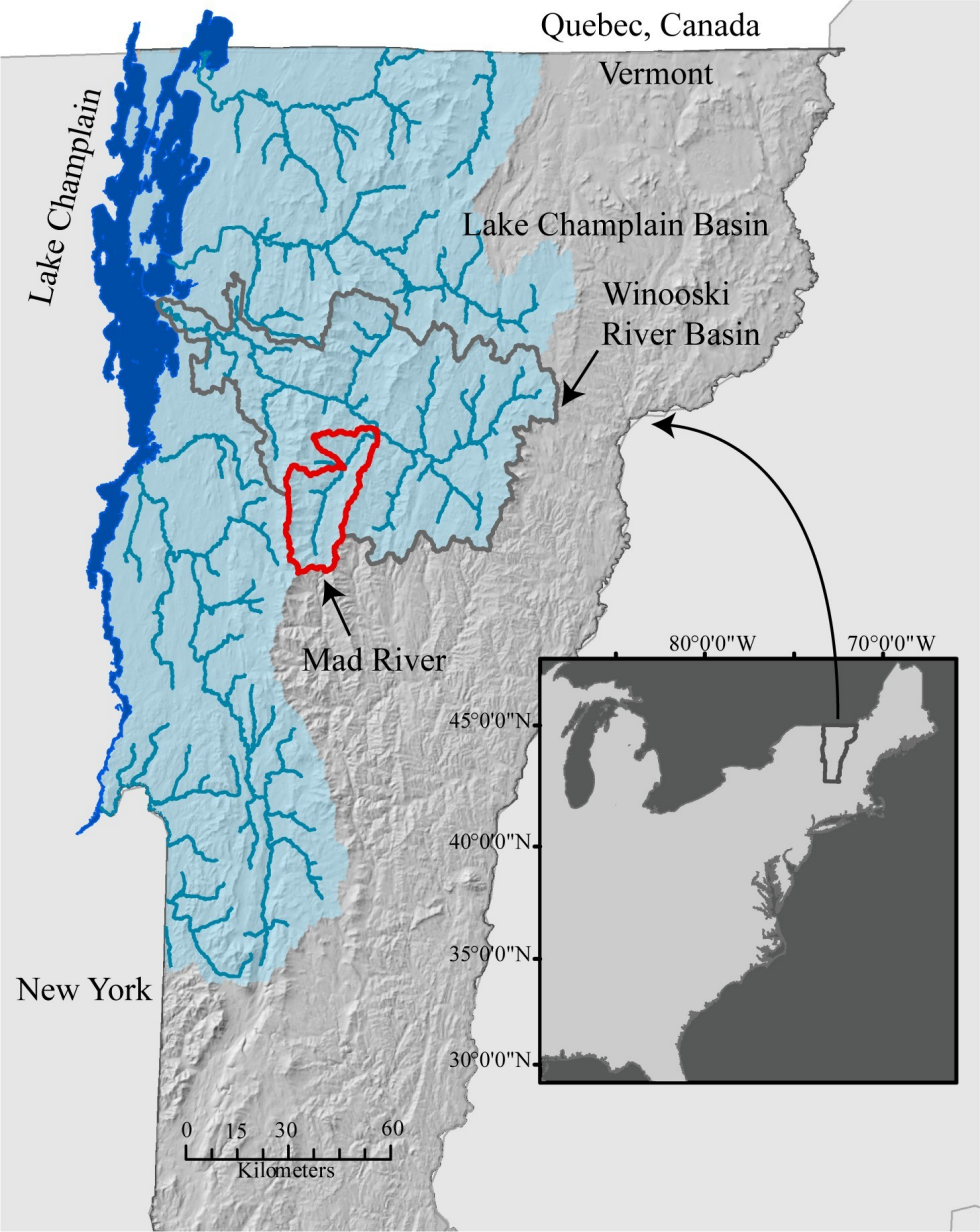
Area of probHAND model application; Lake Champlain Basin, Vermont, USA. We executed the model for 2,083 river-km within 93 different HUC12 units. Model integrity was evaluated from a 1D hydraulic model of three HUC12’s in the Mad River Valley and from 42 observed high water marks within the Winooski River basin. Spatial datasets in figure are from the Vermont Center for Geographic Information and available for open access download at https://geodata.vermont.gov/.

We applied the probHAND model to the Lake Champlain Basin in Vermont using publicly available datasets (Fig 2). The Lake Champlain Basin is an important cultural and economic regional resource but is generally threatened by degraded watershed conditions. Increasingly, efforts to improve flood resiliency have focused on the services provided by natural infrastructure, including floodplains [39–41]. Yet, stakeholders in the region lack an inventory of existing floodplain surfaces, and the degree to which they are hydrologically connected (or disconnected) to the river channel.

In this section we describe the spatial data and parameter inputs used for the Lake Champlain Basin. We executed the model at the scale of the HUC12, applying it to the 93 HUC12’s (and 1172 NHDplus reaches for a total of 2,083 km) that fall completely within Vermont. We evaluated the integrity of probHAND model outputs using predictions from a 1D hydraulic model. Additionally, we explore the sensitivity of model outputs to parametric uncertainty.

### 3.1 Methodology

#### 3.1.1 Model runs and inputs

We executed the probHAND model for the Lake Champlain Basin in Vermont using a 1 m hydro-flattened DEM derived from LiDAR datasets collected between 2013 and 2017, all with a QL2 rating (i.e., vertical accuracy of at least 9.25 cm [42]). We defined our river network to include streams with a drainage area of 25 km^2^ or larger and specified the flow accumulation threshold as such (~2.3x10^7^ pixels). Stream reach extents were delineated using the medium-resolution National Hydrograph Dataset Plus (NHDplus; mapped at a 1:100,000 resolution) clipped to include reaches with a drainage area of 25 km^2^ or greater, based on the mean drainage area associated with each NHDplus reach. Median reach length was 0.6 km, varying greatly in the study area from 4 m to 24 km. Channel slopes extracted from the LiDAR-derived DEM technically represent the water surface slope related to the discharge at the time the LiDAR was collected, since the DEM does not include channel bathymetry. We used a 1m resolution LULC dataset created using 2013–2017 LiDAR and 2016 NAIP imagery for the State of Vermont, available at Vermont’s geodata portal (https://geodata.vermont.gov). We assigned Manning’s *n* values to each land cover type based on calibrated *n* values from a 2D hydrodynamic model of the Otter Creek, Lake Champlain Basin [43]. Flood frequency information was obtained for each reach from USGS’s StreamStats online application. For Vermont, this information is derived from a regression that links drainage area, percentage of lakes and ponds in upstream basin, and horizontal and vertical coordinates to the peak discharge for specific recurrence intervals [44].

To define PDFs for the parameter uncertainty analysis, we compared the calculated baseline values to values derived from other hydraulic or regression models or from measured field data. We identified four PDFs (hydraulic geometry, roughness coefficient, energy grade slope, and peak discharge) and applied them to five input parameters (*A*_*XS*_, *R*_*H*_, *n*_*W*_, *S*, *Q*_*RI*_*)*. We assume a normal distribution for all PDFs and truncate the distributions of energy grade slope and peak discharge distributions at one standard deviation (Table 2). See S1 Text for a description of the methods used to define the PDFs.

**Table 2:** Description of PDF’s specified for Monte Carlo uncertainty analysis in Lake Champlain Basin application.

PDF	Mean	Standard Deviation	Type of Distribution	Source
Energy grade slope	-0.20 * base *S*	0.49 * base *S*	Truncated Normal, 1 SD	HEC-RAS model comparison*
Roughness coefficient	base *n*_*w*_	0.25 * base n_w_	Normal	HEC-RAS model comparison*
Hydraulic geometry	1.16 * base *A*_*xs*_ 1.16 * base *R*_*h*_	0.10 * base *A*_*xs*_ 0.10 * base *R*_*h*_	Normal	LiDAR-USGS Stream Gage comparison**
Peak discharge	base *Q*_*RI*_	SE from flood frequency analysis	Truncated Normal, 1 SD	USGS StreamStats (Olson 2002)

*See S1 Text for a description and S2 Table for models used.

** See S1 Table for stream gage summary.

#### 3.1.2 Probabilistic model integrity and interpretation

We evaluated the integrity of probHAND model outputs using two lines of evidence. Together, these two analyses provide insight into the general capacity of the model to mimic predictions using more robust representations of physical processes and provide context for the mapped distribution of probabilistic flood events.

**Fig 3:**
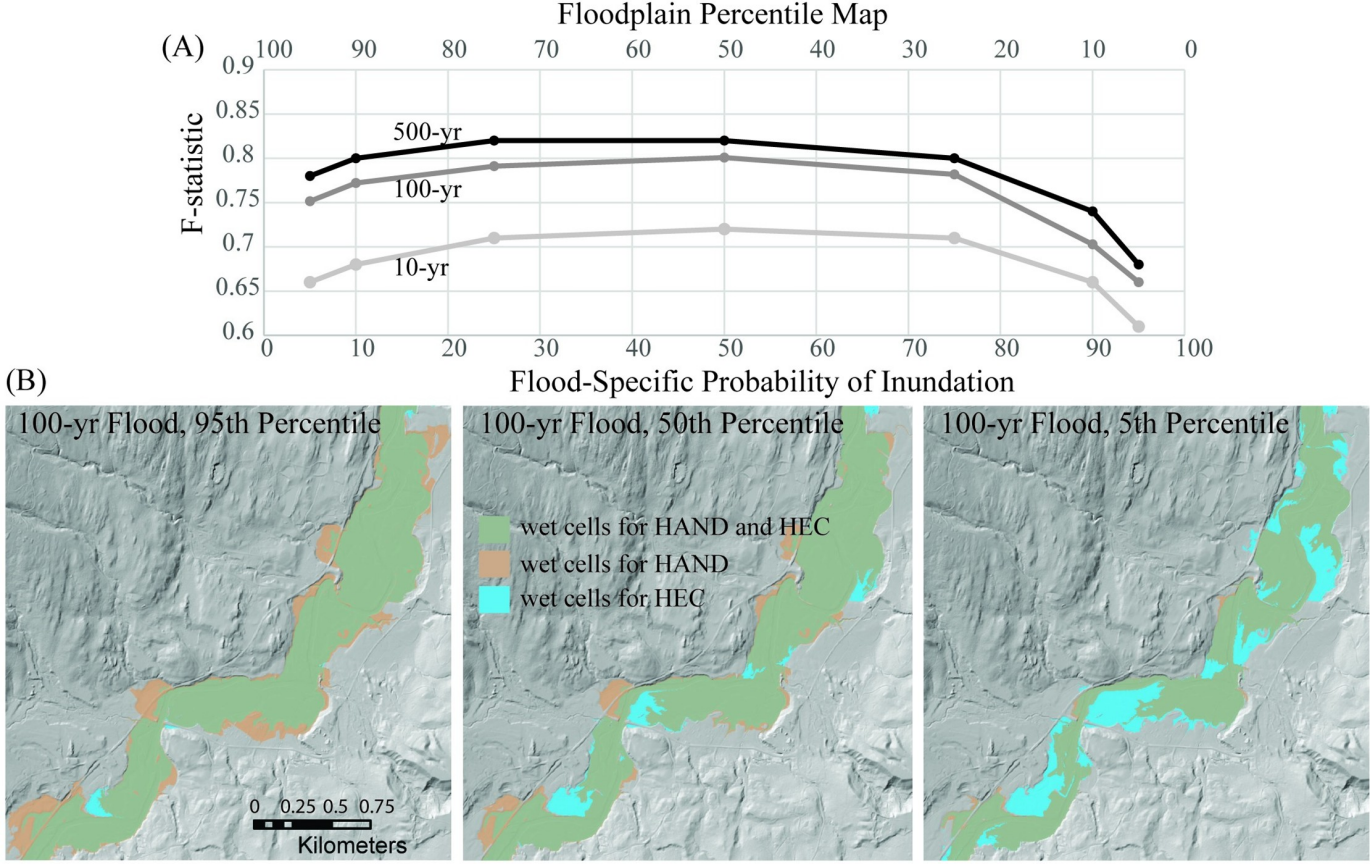
Model evaluation I. Evaluation of integrity of probabilistic floodplain maps from probHAND model (“HAND”) compared to 1D hydraulic model (“HEC”) output for recurrence interval floods. (A) We found that floods with higher recurrence intervals had higher F-statistics (measure of model agreement where 1.0 is perfect agreement) and that 50% probability maps best mimicked hydraulic model output. (B) Example comparison of HAND and HEC models for 100-year flood. Spatial datasets in figure are from the University of Vermont’s Spatial Analysis Lab.

First, we explored the level of agreement between predicted flood extents from a hydraulic model and probHAND model outputs. We used a 1D HEC-RAS model of the Mad River Valley [45], which includes 18 NHDPlus reaches within three HUC12’s (Fig 2). This model was developed using a combination of LIDAR-derived topography and surveyed cross-sections to provide the communities of the Mad River Valley with inundation data and as a tool to support future analyses. A high water mark (HWM) survey dataset associated with a ~500-year flood that occurred in 2011 in the Mad River Valley (see below) and a USGS stream gage were used for model calibration. HEC-RAS model output matched the USGS rating curve well (<0.1 m difference) and the agreement with HWMs was better at the upstream side of bridges (avg: -0.35m difference) than the downstream side (avg: -1.3 m difference) [46]. We compared the steady state result associated with the peak discharge of the 10, 100, and 500 year floods to the range of probable flood extents identified by the probHAND model. This required us to update the peak discharge in the existing HEC-RAS model to match the baseline values used in the probHAND model, and re-run HEC-RAS with the updated hydrologic inputs. HEC-RAS-predicted water surface elevations were used to create floodplain maps using the 1 m DEM’s used as inputs into the probHAND model. To quantify the level of agreement between the HEC-RAS and probHAND floodplain maps, we calculated the Fitness-statistic (F; [20]):

$$F=\frac{C_{HAND+HEC}}{C_{HAND}+C_{HEC}-C_{HAND+HEC}}$$ [4]

where *C*_*HANDw+HECw*_ is the total number of cells predicted wet by both models, *C*_*HANDw*_ is the number of cells predicted to be wet by the probHAND model but either wet or dry by the HEC-RAS model, and *C*_*HEC*_ is the number of cells predicted to be wet by the HEC-RAS model but either wet or dry by the probHAND model (Fig 3). Unlike many other model comparison metrics, the F statistic reduces bias introduced by the choice of model domain, (i.e., the extent of the non-floodplain surface) by considering only the number of conforming wet cells predicted by both models. We compared each of the three recurrence interval floods to the range of percentile maps created by the probHAND model, from 95^th^ to 5^th^ percentiles. We performed an identical analysis of the agreement between the HEC-RAS model’s 100-year flood and FEMAs flood insurance rate map, and the probHAND models 100-year flood (50^th^ percentile) and the FEMA map.

**Fig 4:**
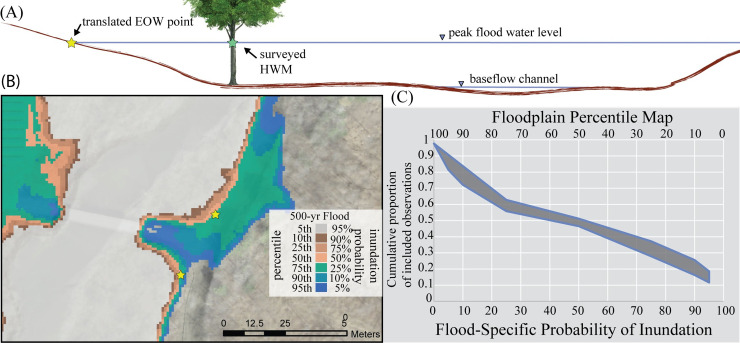
Model evaluation II. Evaluation of performance of probHAND probabilistic floodplain maps relative to surveyed high water marks. (A) First, we translated high water marks (HWM) to edge of water (EOW) locations. (B) We then identified the highest probability map, for the approximate recurrence interval flood, which encompassed the EOW point. (C) We found that floodplain map probabilities well-represented the distribution of observed flood extents. Cumulative plot of HWM observations that increasingly fall within lower probability (higher percentile) maps. The band represents uncertainty in the location of the HWM observations. Spatial datasets in figure are from the University of Vermont’s Spatial Analysis Lab.

We also compared observations of flood inundation extents from a large flood to the distribution of probable flood extents from the probHAND model. We used 42 high water marks (HWM) surveyed following Tropical Storm Irene in August 2011 within the Winooski River Basin (Fig 4), which resulted in significant flooding (e.g., 50–500 year flood; [46]). To do this, we first converted the HWM into an edge of water (EOW) observation by identifying the onshore ground surface that intersected the surveyed elevation above the ground of the HWM point (Fig 4). Next, we identified the recurrence interval of the peak discharge of Irene at each HWM observation using a relationship between peak discharge recorded at USGS stream gages (and translated to a recurrence interval using USGS StreamStats Data-Collection Station Report) and drainage area. Finally, we identified the lowest percentile band that encompassed the edge of water. We calculated the range of potential values, given uncertainties in the elevation of the EOW and in the peak discharge recurrence interval along ungaged streams.

#### 3.1.3 Contribution to variance

We estimated the extent to which uncertainty in each parameter contributes to variance in floodplain model output. Outputs of the Monte Carlo simulation were used to fit multivariate linear models relating stage (i.e., HAND elevation) (*Y*) to the sampled parameters (*X*_*i*_). Five variables (*A*_*xs*_, *R*_*H*_, *S*, *n*_*W*_, *Q*_*RI*_) are input to the model to determine Y. We took the square root of slope to conform to a linear regression model. Because *A*_*xs*_ and *R*_*H*_ are highly correlated and their uncertainty determined from the same distribution (see S1 Text), we combined them into a new variable (*geom*):

geom=ln((AXSRH)35) [5]

We obtained the standardized regression coefficients *β*_*i*_^*2*^ by normalizing the slopes *b*_*i*._

$$Y=\sum b_{i}X_{i}$$ [6]

$$\beta i=b_{i}\frac{\sigma_{X_{i}}}{\sigma_{Y}}$$ [7]

The standardized regression coefficients are a valid measure of contribution to variance if the coefficient of determination R^2^ is higher than 0.7, representing the first-order contribution to variance of the variable *X*_*i*_ to *Y*. We repeated this analysis for each of the 1172 reaches and 8 recurrence interval floods.

We explored the impact of the width of probability distributions on the sensitivity analysis by repeating the analysis on a subset (+/- 1 standard deviation) of the 1000 values of each sampled parameter. This resulted in a subset of 680 values for each parameter, and a total of 240 simulations. We refer to the results based on the subsampled distributions as the “narrow distribution” and the results based on the original distributions, with the full 1000 simulations, as the “wide distribution.”

We hypothesize that the importance of each variable will shift based on characteristics of the river and valley itself. To explore this additional factor, we use reach slope to classify all reaches into three valley setting groups. Except for *n*_*W*_ between groups 1 and 2, the variables used in the contribution to variance analysis are statistically different among the three groups (Table 3).

**Table 3:** Average parameter values for valley setting groups, classified by reach slope.

	2-year flood				100-year flood			
Group	*geom*	*S*	*n*_*W*_	*Q*_*RI*_	*geom*	*S*	*n*_*W*_	*Q*_*RI*_
1	11.2	0.0004	0.076	251	13.7	0.0004	0.080	824
2	7.1	0.003	0.075	111	9.6	0.003	0.081	381
3	5.0	0.014	0.089	55	7.7	0.014	0.093	194

### 3.2 Results

#### 3.2.1 Probabilistic HAND maps for the Lake Champlain Basin

**Fig 5:**
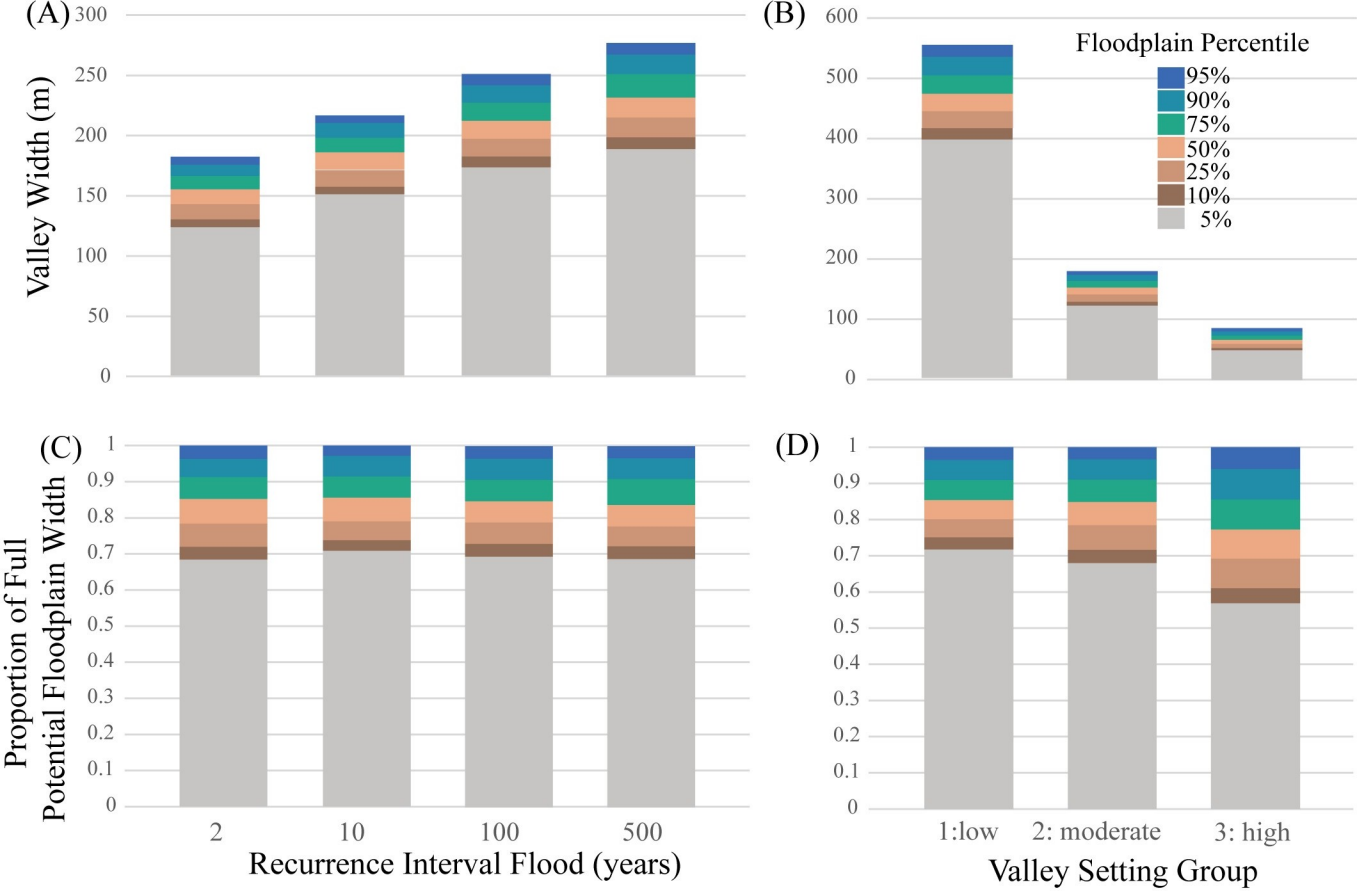
Floodplain width. Summarized by recurrence interval flood (A&C) and valley setting, for the 100-yr flood (B&D) in the Lake Champlain Basin, Vermont. Floodplain width is calculated as the total mapped area divided by the total stream length (A&B), and greatly differs among valley setting groups (B). The proportional increase of the total potential width (i.e., the 95^th^ percentile map) does not differ substantially for floods of varying magnitude (C) but does differ based on valley setting (D). Uncertainty in input parameters translates to larger differences in floodplain width in settings with high channel slope that have narrower valleys, than for floodplains in settings with either low or moderate channel slopes.

We mapped ~400 km^2^ of floodplain along 1,700 km of rivers in Vermont. On average for the Lake Champlain Basin, VT, 95^th^ percentile maps have floodplain widths of 124 and 188 m, for the 2- to 500-year floods, respectively (Fig 5A). By expanding the probability of inundation to include areas with a low, but real, chance of inundation (i.e., 5^th^ percentile), the full width of potential floodplain areas increases by 30–32% (depending on the magnitude of the flood event), adding 57 and 87 m of floodplain width to the 2- and 500-year floods, respectively. Along approximately 15% of the targeted streams, the presence of a reservoir or improper hydro-flattening of the DEM caused errors in the floodplain mapping process [47]. We removed these areas from the presentation of results.

Depending on valley setting, however, floodplain width varies greatly, on average ranging from 48 m for the 5^th^ percentile of the 100-year flood in settings with steep slopes (group 3) to 397 m in settings with low slopes (group 1; Fig 5C). By expanding the probability of inundation to include areas with a low, but real chance of inundation for the 100-year flood (i.e., 95^th^ percentile), increases floodplain width as a proportion of the high probability width (i.e., 5^th^ percentile) to a greater extent in settings with steep slopes (43%) than in those with low or moderate slopes (28%; Fig 5D).

#### 3.2.2 Model integrity and interpretation

We found good agreement between probHAND probability distributions and 1D HEC-RAS maps (Fig 3), with F-statistics ranging between 0.61 and 0.82 and averaging 0.74 for the 10-, 100-, and 500-year floods. Agreement between the two models increased with increasing flood recurrence and is greatest for the 500-year flood. For all recurrence intervals, the greatest number of conforming wet cells was associated with the 50% probability maps, and a minimum for the most conservative maps (i.e., those that represented areas with a 90–95% probability of inundation). Agreement between the HEC-RAS and probHAND model output for the 100-year flood was better (50^th^ percentile, 0.80) than between the probHAND model and FEMA map (0.75) or the HEC-RAS output and FEMA map (0.69).

The probability of inundation identified by the probHAND model captures the majority of field documented high-water marks (Fig 4). Out of the 42 points we evaluated in the Winooski River Basin, only ~10% were not included in the range of probable floodplain extents produced by probHAND maps. More so, we found that the distribution of field observed flood extents was well-described by the distribution of floodplain extent probabilities. About 20% of the observed points were shown to be within the floodplain by the conservative maps and 50% of the points by the 50% probability maps.

#### 3.2.3 Contribution to variance

**Fig 6:**
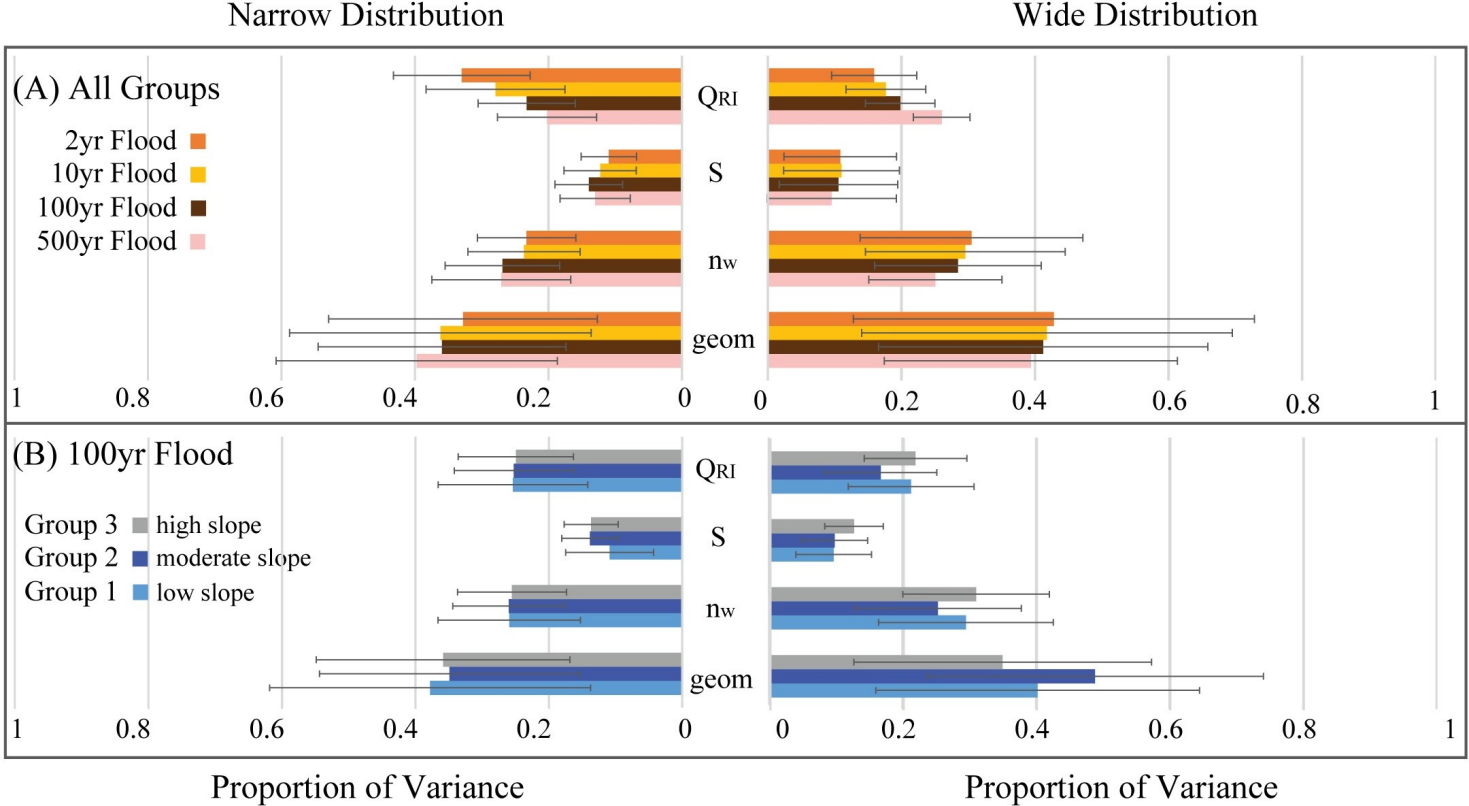
Contribution to variance. The proportion of variance in model output (predicted stage for a given recurrence interval flood) described by four input parameters, based on the original distribution (right) and a narrower subset (left). (A) Although the proportion contributed by *geom* decreases with increasing flood magnitude and the proportion of variance contributed by *Q*_*RI*_ increases, the contribution of each parameter remains relatively similar (e.g., *geom* is greatest and *S* is least). Narrowing the distribution also reduces the variability contributed by *geom* while increasing *Q*_*RI*_. (B) Valley characteristics mediate the contribution to variance, which is also influenced by the width of the probability distribution.

Hydraulic geometry, represented by the variable *geom*, contributes the largest proportion of variance in inundation stage (~40%), while channel slope describes the smallest proportion (~10%) (Fig 6). With increasing flood magnitude and a narrower uncertainty distribution, the proportion of the variance described by hydraulic geometry decreases, while discharge (*Q*_*RI*_) increases, although the ranking of the four variables remains the same. Weighted n (*n*_*w*_) and channel slope (*S*) describe less of the variance for increasingly larger flood events, but these variables describe a greater proportion of the variance with a narrow uncertainty distribution when compared to the wide distribution.

Valley setting also influences the proportion of variability described by the four input variables (Fig 6). For river valleys with low *S*, which also generally have larger channels (i.e., large *geom* values), greater *Q*_*RI*_, and lower *n*_*w*_ values (i.e., Group 1), the importance of *S* is less (~7%) than for other settings, increasing in importance with increasing *S*, (e.g., to Group 2 and Group 3). The importance of the remaining variables is also influenced by valley setting, especially when uncertainty distributions are wide.

## 4. Discussion

### 4.1 Utility of the probHAND model for communicating flood hazards

In this paper, we describe a relatively rapid approach to identifying probabilistic distributions of inundating flood extents and demonstrate the application to a large area in Vermont, USA. Our mapping approach, while simple in its representation of process, produces floodplain maps with a high rate of agreement to those created with a hydraulic model, which has a more robust representation of process, but is data intensive and has greater computational demands. As such, our work supports the assessment that a new generation of floodplain mapping approaches, notably those classified as low-complexity, has great promise to improve upon our understanding and communication of flood risk and conservation practices [18,20,24,48].

Currently, flood risk management in the United States largely relies on publicly available flood maps (DFIRM) developed by FEMA in support of the National Flood Insurance Program [49]. Often, DFIRM’s are outdated, not reflective of recent shifts to environmental and landscape conditions (e.g., urbanization, intensifying precipitation patterns), or have incomplete spatial coverage [13]. They also convey a limited amount of information on flood hazards (i.e., typically only that of the 100 year flood; [12]). But modifying, extending, and updating DFIRM’s is difficult due to institutional and implementation challenges [5].

We believe that the probHAND model and the resulting probabilistic floodplain maps may provide an alternative reference for flood risk management. In its current form, the probHAND product is unlikely suitable to support the NFIP and the determination of flood insurance rates. Although the probHAND had better agreement with the calibrated hydraulic model, than did the DFIRM, documented local uncertainties, both in our model and elsewhere, are likely too high to identify the flood risk for a single property [24,50]. It may, however, provide a good resource for other flood risk management applications, including public education and emergency planning, notably over regional scales. The resulting probabilistic maps may also support riparian conservation planning by identifying opportunities for floodplain restoration or protection [51].

With an open-source code, accessible inputs, and robust representation of the probability of flood inundation for floods with a large range of frequencies, states, municipalities, or other stakeholders could execute the model. Additionally, floodplain maps may be amended relatively easily to shifts in inputs from either an improved understanding of process, uncertainty, or current or future changes, although updates to the model code may enhance the functionality of such evaluations (e.g., [52]).

Deterministic representation of flood extents, such as the DFIRM maps, can provide a false sense of precision, masking potentially large uncertainties involved in identifying the distribution of floodplains across the landscape [30]. Inaccuracies in mapped flood extents from low-complexity models may be particularly large in urban settings or at confluences where simple process representations do not capture local hydraulic conditions [20,24] and greatest for floods with smaller peak magnitudes, which are more heavily influenced by local topographic and hydraulic conditions than large floods.

By including an uncertainty analysis into the modeling framework, the resulting probabilistic maps communicate not only the likelihood of inundation for a given flood event, but also the level of (un)certainty. In the probHAND model, these uncertainties are associated with errors in the measurement or representation of input parameters, such as channel hydraulic geometry, and in process uncertainties, such as the assumption of uniform flow described by the Manning’s equation. We found that probabilistic maps capture the distribution of uncertainty within a dataset of field observations of flood extents, and from calibrated hydraulic model output. We interpret these results to suggest that with the incorporation of uncertainty, the probHAND model fulfills its intended purpose, providing a way to evaluate the extent of flooding to communicate flood risks. Thus, additional complexity may not be needed in models with similar goals [53].

Uncertainties translate to on-the-ground changes to inundated areas, or floodplain widths, the degree to which depends on valley setting. In valleys with low slopes, uncertainty results in large absolute increases in width and in valleys with steep slopes, uncertainty results in large relative increases in width. As such, the uncertainty of risks associated with flooding differs amongst communities in varying geographic settings, and these differences must be accounted for in educating the public and developing a flood risk management plan [49].

### 4.2 Investments in improving flood hazard map accuracy

With rapid improvements to the availability and quality of remotely-sensed data, increasing computation efficiencies, and innovations in modeling procedures, barriers to the development of floodplain map datasets for large areas no longer exist [54]. At large scales, however, there are limited options for calibration. As such, reliability of this new generation of maps requires accurate measurement of parameter inputs and their associated uncertainty [48]. But refinement of remotely acquired input values, from field measurements, for example, is expensive and time intensive. Instead, limited resources should be invested into constraining those variables that contribute the most to variability in modeled floodplain extent.

Model output variability is most sensitive to uncertainty in hydraulic geometry. This suggests that improvements to the accuracy of the underlying topography, from which reach-average hydraulic geometry measurements are extracted, should be a priority [47]. Availability of high-quality topographic data (i.e., supporting <10 m resolution DEM’s) for the United States is growing, increasing the opportunity for probHAND models to use high resolution DEMs. DEM resolution influences HAND map values, and therefore inundation depths and extents, especially for smaller stages [55], in tighter valleys where channels are small, and in urban areas [31]. Typically, higher resolution DEMs more closely mimic complex model output or field observations [31,55]. Where feasible, we recommend investments into more accurate topographic data. However, high resolution datasets may become financially and computationally prohibitive, and their impact on floodplain map accuracy may be limited if large uncertainties remain for other parameters [22,56,57]. Additionally, various sources of error may be introduced during pre-processing of the DEM for creation of the HAND layer, including channel extraction, which can increase uncertainty in the resulting floodplain maps. Alternative, and additional, DEM processing steps have recently been proposed [18,47] and should be considered, and their benefits weighed, in light of the added computational resources.

Often, DEMs do not include below-water topography and the importance of incorporating channel bathymetry in the general HAND modeling approach, compared to the general uncertainty associated with hydraulic geometry measurement error, remains inconclusive [20,21]. Improvements to the representation of channel bathymetry may take the form of simple proportional increases in hydraulic geometry values, as we have done in the Lake Champlain application, empirically derived updates [e.g., 20], or merging field measured bathymetry.

Flood frequency analyses that define discharge inputs in the probHAND model have a high degree of uncertainty, especially on ungaged streams [58], and this uncertainty contributed anywhere from approximately 15% of variability in model output for small floods (i.e., 2 yr recurrence) to 40% for very large floods (i.e., 500 yr recurrence). Uncertainty in discharge is likely to continue to increase as runoff patterns shift in response to a changing climate [1]. In some regions, such as the Lake Champlain Basin, large floods are becoming more common, further increasing the importance of limiting uncertainty [59]. Regression or similarity-based regionalization techniques are commonly used to identify flood quantiles. Methodological improvements (e.g., [38]) or enhancements to the underlying datasets, including expansion of the stream gaging network [60], may help to better constrain peak flood discharges associated with specified flood magnitudes.

The influence of uncertainty of the Manning’s roughness coefficient on model output variance is generally comparable to discharge, representing 20–40% of the variability in predicted stage. Increasingly sophisticated methods are being developed to more accurately constrain landcover-specific roughness values [61,62]. Yet, many have questioned the applicability of spatially distributed roughness parameterization to floodplain mapping models [25]. In hydraulic and hydrodynamic models, roughness values serve as tuning parameters and their values depend on process simplifications and code structure [63]. Robust calibration datasets (e.g., 2D hydraulic models, extensive field surveys of flood water extents of various flood events, or satellite imagery), may be used to constrain roughness values, and may also be used to more precisely represent slope [47]. The creation, or purchase, of such datasets can require significant resources, and may not be useful in all settings (e.g., satellite imagery is often too coarse for small rivers).

More thoughtful reach delineations may also reduce uncertainty [47,50]. While short reaches are often not long enough to adequately represent slope [50], longer reaches could mask important valley and channel morphology. Numerous segmentation approaches have been proposed (e.g., [64] and may be adopted to a watershed, or region to create geomorphically-consistent reaches. Additionally, reaches of a uniform length have been found to improve HAND model accuracy [47].

Overall, investment made towards limiting all parametric uncertainty, which has the effect of narrowing PDFs, has the general impact of reducing the influence of hydraulic geometry while increasing the influence of discharge. Yet, the ranking of each variable remains the same. We recommend that with limited financial, human, and computation resources, users of the probHAND model make investments to better constrain hydraulic geometry and discharge values. Where feasible, this includes investing into more accurate, higher resolution topographic datasets, especially ones that are carefully hydro-flattened, and a better understanding of the relationship between the frequency and magnitude of flood events on ungaged basins. Practitioners adopting the probHAND model to their region should also keep an eye out for opportunities to constrain hydraulic geometry and water surface slopes through, for example, existing field surveys. Additionally, accurate representations of channel locations and reach breaks will help to improve overall model performance.

## 5. Conclusion

The probHAND model presented in this paper formalizes and extends the functionality of the HAND floodplain mapping approach by accounting for uncertainty associated with measurement error and process simplification. Using publicly available data and open source code, the probHAND model may be applied over large scales, in a relatively rapid fashion. The resulting probabilistic maps help communicate flood hazards and may also be used to prioritize and evaluate the impact of floodplain restoration measures, delineating areas that are likely to be wet during, say the 100-year flood from those that are dry, and the uncertainty associated with such delineations. Uncertainty in flood stage translates to a range of flood extents, and for a large flood event in the Lake Champlain Basin, observed high water marks fell within this range. This observation suggests that probabilistic approaches may make up for deficiencies in low complexity models (i.e., their inability to capture complex hydraulic settings). As such, low-complexity models, rather than more complex data and resource intensive alternatives (i.e, hydraulic models), that incorporate uncertainty may be sufficient for flood risk management and conservation applications. Constraining uncertainty, however, can improve model accuracy and investments into high quality topographic data, and more precise flood frequency analyses will reduce model output variability. The probHAND model may easily be adapted to explore how changing input parameter values alter inundation patterns, a powerful tool in the face of shifting precipitation and land use patterns, as the climate changes and populations grow.

## Supporting information

S1 Table List of USGS stream gage metrics. Summary of comparison between LiDAR-derived cross-sections and those measured at USGS stream gages used to derive values for the hydraulic geometry PDF.
(DOCX)

S2 Table One-dimensional hydraulic models. Summary of observations extracted from 1D HEC-RAS models used to compare probHAND model-derived slopes to define slope PDF.
(DOCX)

S1 Text Calculating uncertainty for input parameters to Lake Champlain Basin, Vermont Application. (DOCX)
