# Supplementary material for: Improving flood hazard datasets using a low-complexity, probabilistic floodplain mapping approach
Source: PLoS One. 2021 Mar 29;16(3):e0248683. doi: 10.1371/journal.pone.0248683 (PMC8006981; doi:10.1371/journal.pone.0248683)
Supplement: S1 Table — Summary of comparison between LiDAR-derived cross-sections and those measured at USGS stream gages used to derive values for the hydraulic geometry PDF. (DOCX) [file pone.0248683.s001.docx]

**S1 Table. List of USGS stream gage metrics.** Summary of comparison between LiDAR-derived cross-sections and those measured at USGS stream gages used to derive values for the hydraulic geometry PDF.

| **USGS Stream Gage Name** | **HAND Slope at Gage** | **Drainage Area at Gage (km^2^)** | **% of Q_2_ Flow Area Underestimated by LiDAR** | **Median Q during flight as % of Q_2_** | **LiDAR Flight Date** |
| --- | --- | --- | --- | --- | --- |
| Pike River at East Franklin | 0.0020 | 89 | 14.0% | 3.6% | Nov 8-21, 2017 |
| LaPlatte River near Shelburne Falls | 0.0029 | 116 | 9.7% | 2.8% | Nov 8-11, 2014 |
| Little Otter Creek at Ferrisburgh | 0.0034 | 148 | 10.8% | 2.1% | Nov 8-21, 2017 |
| North Branch Winooski at Wrightsville | 0.0040 | 179 | 30.1% | 3.8% | Oct 27, 2015 |
| Dog River at Northfield | 0.0045 | 199 | 10.5% | 0.9% | Oct 27, 2015 |
| Lewis Creek at Ferrisburgh | 0.0038 | 200 | 11.2% | 2.8% | Nov 8-21, 2017 |
| New Haven, Brooksville | 0.0027 | 298 | 25.4% | 2.9% | Nov 8-21, 2017 |
| Missisquoi at Troy | 0.0057 | 339 | 5.5% | 4.3% | May 9, 2015 |
| Mad River Moretown | 0.0023 | 360 | 19.7% | 8.1% | May 7, 2015 |
| Otter, Rutland | 0.0055 | 795 | 4.3% | 2.9% | Nov 8-21, 2017 |
| Winooski River at Montpelier | 0.0010 | 1028 | 9.7% | 18.0% | Oct27, 2015 |
| Little River near Waterbury | 0.0130 | 1108 | 36.5% | 9.8% | May 8-9, 2015 |
| Missisquoi near East Berkshire | 0.0005 | 1241 | 23.9% | 2.9% | Nov8-21, 2017 |
| Lamoille River at Johnson | 0.0023 | 1312 | 4.7% | 18.9% | Oct 24, 2015 |
| Otter, Middlebury | 0.0030 | 1626 | 34.7% | 6.0% | Nov 8-21, 2017 |
| Lamoille River at East Georgia | 0.0001 | 1777 | 12.0% | 8.3% | Nov 8-11, 2014 |
| Missisquoi at Swanton | 0.0003 | 2201 | 18.8% | 4.5% | Nov 8-21, 2017 |
| Winooski near Essex Junction | 0.0021 | 2704 | 9.9% | 3.6% | Nov 8-11, 2014 |
