# Supplementary material for: Improving flood hazard datasets using a low-complexity, probabilistic floodplain mapping approach
Source: PLoS One. 2021 Mar 29;16(3):e0248683. doi: 10.1371/journal.pone.0248683 (PMC8006981; doi:10.1371/journal.pone.0248683)
Supplement: S2 Table — Summary of observations extracted from 1D HEC-RAS models used to compare probHAND model-derived slopes to define slope PDF. (DOCX) [file pone.0248683.s002.docx]

**S2 Table. One-dimensional hydraulic models.** Summary of observations extracted from 1D HEC-RAS models used to compare probHAND model-derived slopes to define slope PDF.

| **Model** | **# of NHDplus Reaches in Model** | **# of Observations** | **HAND stages** | **HAND slopes** | **Modeled Slopes** | **Modeled Q Range (m^3^/s)** |
| --- | --- | --- | --- | --- | --- | --- |
| Mad River | 17 | 123 | 1.1 - 5.9 | 0.0018 - 0.018 | 0.0005 - 0.023 | 17 - 911 |
| Browns River | 53 | 224 | 1.2 - 5.2 | 0.0005 - 0.028 | 0.00001 - 0.010 | 35 - 235 |
| Stevens Brook | 5 | 32 | 2.5 - 6.5 | 0.0023 - 0.015 | 0.0004 - 0.007 | 142 - 512 |
| Winooski River | 19 | 84 | 2.8 - 11.2 | 0.0002 - 0.044 | 0.00001 - 0.002 | 730 - 2176 |
