# Supplementary material for: Improving flood hazard datasets using a low-complexity, probabilistic floodplain mapping approach
Source: PLoS One. 2021 Mar 29;16(3):e0248683. doi: 10.1371/journal.pone.0248683 (PMC8006981; doi:10.1371/journal.pone.0248683)
Supplement: S1 Text — (DOCX) [file pone.0248683.s003.docx]

***Calculating Uncertainty for Input Parameters***

To define the hydraulic geometry PDF, applied to *A_XS_* and *R_H_*, we compared the baseline cross-sectional area measurements to detailed cross-section measurements collected at 18 USGS stream gages located within the Lake Champlain study area (S1 Table). Because the DEM for Lake Champlain was built from LiDAR, hydraulic geometry measurements do not include below water bathymetric topography. Thus, baseline hydraulic geometry values underestimate true values. To estimate measurement errors, we first identified the channel bottom elevation from the HAND layer and associated that elevation with the gage datum (reported in feet above NGVD29). Using reported stream gage measurements, and filtering for rating (only fair or good) and location (using a consistent location), we quantified the cross-sectional area below the water surface at the time of the LiDAR flight. We report those values as the percent of the corresponding NHDplus reaches’ *Q_2_* cross-sectional area (S1 Table), and use the average value (16%) and standard deviation (10%) of these values to define the PDF for hydraulic geometry.

To define PDFs for energy grade slope and roughness coefficient, we compared baseline values to those calculated by, or calibrated for, four 1D HEC-RAS models in the basin, respectively (S2 Table). We matched HEC-RAS cross-sections with NHDplus reaches, and compared *S* and *n_w_* values for the same discharges. When multiple cross-sections occurred within a single NHDplus reach, we took the average for each modeled discharge value. In total, we made 463 comparisons that ranged between 17 and 2176 m^3^/s and energy grade slopes between 0.0001 and 0.023. We found that baseline slope values over-estimated modelled energy grade slopes, by on average 20% (SD 49%). The range of *n_w_* values calculated by the probHAND model was much greater than those used in the HEC model (average difference of 35%, SD of 40%) and when compared to the HEC-RAS values, the calculated baseline values were systematically underestimated; for increasing baseline *n_w_* values, the difference between the baseline values and the HEC-RAS values increased. Because roughness coefficients are typically used to calibrate hydraulic models, comparison of values between two types of models may not be straightforward (57). Thus, we did not use the HEC-RAS data to define the roughness coefficient PDF. Instead, we based our distribution on typical ranges of values reported in the literature (0% mean, 25% SD; e.g., (60). Finally, we used the standard error of residuals for each regression equation to define the PDF for peak discharge (40).
